# An attempt to interpret a biochemical mechanism of C_4_ photosynthetic thermo-tolerance under sudden heat shock on detached leaf in elevated CO_2_ grown maize

**DOI:** 10.1371/journal.pone.0187437

**Published:** 2017-12-08

**Authors:** Mingnan Qu, James A. Bunce, Richard C. Sicher, Xiaocen Zhu, Bo Gao, Genyun Chen

**Affiliations:** 1 CAS Center for Excellence in Molecular Plant Sciences, Institute of Plant Physiology and Ecology, Shanghai Institutes for Biological Sciences, Chinese academy of Sciences, Shanghai, China; 2 USDA-ARS, Crop Systems and Global Change Laboratory, Beltsville, MD, United States of America; 3 Centralab Institute of Basic Medical Science, Chinese Academy of Medical Sciences, Beijing, China; Northeast Forestry University, CHINA

## Abstract

Detached leaves at top canopy structures always experience higher solar irradiance and leaf temperature under natural conditions. The ability of tolerance to high temperature represents thermotolerance potential of whole-plants, but was less of concern. In this study, we used a heat-tolerant (B76) and a heat-susceptible (B106) maize inbred line to assess the possible mitigation of sudden heat shock (SHS) effects on photosynthesis (P_N_) and C_4_ assimilation pathway by elevated [CO_2_]. Two maize lines were grown in field-based open top chambers (OTCs) at ambient and elevated (+180 ppm) [CO_2_]_._ Top-expanded leaves for 30 days after emergence were suddenly exposed to a 45°C SHS for 2 hours in midday during measurements. Analysis on thermostability of cellular membrane showed there was 20% greater electrolyte leakage in response to the SHS in B106 compared to B76, in agreement with prior studies. Elevated [CO_2_] protected P_N_ from SHS in B76 but not B106. The responses of P_N_ to SHS among the two lines and grown CO_2_ treatments were closely correlated with measured decreases of NADP-ME enzyme activity and also to its reduced transcript abundance. The SHS treatments induced starch depletion, the accumulation of hexoses and also disrupted the TCA cycle as well as the C_4_ assimilation pathway in the both lines. Elevated [CO_2_] reversed SHS effects on citrate and related TCA cycle metabolites in B106 but the effects of elevated [CO_2_] were small in B76. These findings suggested that heat stress tolerance is a complex trait, and it is difficult to identify biochemical, physiological or molecular markers that accurately and consistently predict heat stress tolerance.

## Introduction

The daily, seasonal, and annual mean temperatures experienced by plants have increased as a result of human-caused increase in atmospheric CO_2_ concentration [1]. Accumulative studies have examined interactive effects of temperature and CO_2_ on both C_3_ and C_4_ plants. The interactive effects cover varies of physiological and biochemical aspects, for instance, stomata driven water use efficiency [2–5], leaf morphology [6], photorespiration [7], photosystem II efficiency [8, 9] and Rubisco (ribulose 1,5-bisphosphate carboxylase/oxygenase) activase [10, 11]. In contrast, the interactions between elevated CO_2_ and sudden heat shock (SHS) have been examined in only a few studies [12]. In the field conditions, it has been proved that this type of abnormally extreme weather events also may decrease crop growth and reduce harvestable yields [13]. Studies on the effects of heat stress in C_4_ plants have been well reported [e.g., 4–7, 11, 13]. In general, C_4_ plants have better adaption to warmer climates than C_3_ plants, this may be due to the fact that C_4_ possess special pathway of photosynthetic-carbon metabolism (PCM). However, it remains unclear if elevated [CO_2_] can reprogram the PCM in response to SHS in C_4_ plants, such as maize. Therefore, understanding physiological, biochemical and molecular processes in maize caused by elevated [CO_2_] undeniably facilitates the prediction of plant responses to future climate [11].

Previous studies have shown that the decreases of net photosynthesis (P_N_) after SHS in maize cannot be fully explained by a stomatal limitation because *C*_*i*_ values were sufficiently high to prevent CO_2_ from inhibiting rates of P_N_ [14]. Therefore, it was likely that high temperature effects on P_N_ were the result of impaired metabolic processes in the leaf. Prior evidence suggested that a deactivation of Rubisco is an early event in the inhibition of P_N_ in response to high temperature [15]. Additionally, Rubisco activase, which is a chloroplast protein that is essential for maintaining Rubisco in an active state, may be inhibited by high temperature stress [16]. Consequently, these evidences suggest that high temperatures inhibit the Calvin cycle and reduce the rate of synthesis of ribulose-1,5-bisphosphate, the substrate for Rubisco. On the other hand, it has been argued that the C_4_ cycle is more sensitive to water stress than the Calvin cycle [17, 18], and the same could be true for high temperature stress. The inactivation of P_N_ by high temperatures may also be related to membrane damage within the chloroplast and at various other sites in the cell. For example, increased electrolyte leakage was observed following high temperature treatments, suggesting that the cellular membrane was disrupted by heat stress [19].

Metabolite analysis is an effective method for elucidating mechanisms of abiotic stress tolerance, including heat stress [20]. Mayer et al. [21] reported an increase in the abundance of γ-aminobutyric acid (GABA), β-alanine, alanine, and proline in cowpea (*Vigna unguiculata*) as a result of heat shock. Song et al. [22] also reported that several metabolites in leaves of Kentucky bluegrass (*Poa pratensis*) were accumulated shortly after heat stress treatments. These previous metabolite studies were exclusively performed using ambient CO_2_ and very few studies have investigated the changes of metabolite accumulation in response to SHS under elevated [CO_2_] conditions. Elevated [CO_2_] treatments can have mitigating effects on the response of plant growth to drought stress or nutritional deficiencies [23]. Therefore, it would be valuable to know if elevated [CO_2_] could mitigate the effects of SHS on plant metabolism. The hypothesis in this study was given that elevated [CO_2_] would mitigate SHS effects on maize seedlings, and that the effects would differ in lines with contrasting heat stress tolerance.

## Materials and methods

### Materials and experimental set-up

Two maize (*Zea mays L*.) inbred lines, i.e., B76 (heat tolerant) and B106 (heat susceptible), were used in this study. These two maize genotypes differed with respect to thermo-tolerance based on the polymorphism of several phenotypic markers [24, 25]. Seeds of B76 (PI 550483) and B106 (PI 594049) were obtained from U.S. Germplasm Resources Information Network (GRIN: http://www.ars-grin.gov/).

The experiment was conducted in field-based, open top chambers (OTCs) to examine the heat tolerance of maize cultivars grown under ambient and elevated [CO_2_]. The experimental site was located at the Beltsville Agricultural Research Center, USDA-ARS (39° 00' N, 76° 56' W), Beltsville, MD, USA. Seeds of both inbred lines were sown in six OTCs measuring 2 m long by 1.5 m wide by 2 m high starting from May 24^th^ in 2013. Each chamber was spaced 2 m apart to minimize shading, and individual plants were thinned at 7 days after emergence (DAE) to 15 cm distance. The soil in each OTC was kept moist by applying water once weekly to field capacity. Plants in OTCs were exposed to ambient air or ambient air plus 180 ppm CO_2_ as described elsewhere [26]. There were three chambers per CO_2_ treatment, and all chambers were planted with both maize inbred lines. Mean and maximum air temperatures were 23.8 and 37.6 ^o^C, respectively, during the period when experiments were performed. Mean daytime CO_2_ concentrations were 394 and 566 ppm, in the ambient and elevated OTC chambers, respectively.

### Heat stress treatments and gas exchange measurements

Sudden heat shock (SHS) treatments were applied to individual leaves between 10:00 am and 12:00 pm on six clear sunny days in 2013. The treatments were applied when the sixth leaf was fully expanded about 30 days after emergence (DAE). Leaf gas exchange rates were measured on the marked sections of the leaves using a CIRAS-1 Portable Photosynthesis System (PP system, Amesbury, MA). After initial leaf gas exchange measurements, marked sections of intact leaves were placed in water-jacketed leaf cuvettes with an internal radiator and fan. Air temperatures within the cuvettes were raised to 45 ^o^C by circulating heated water from a temperature controlled bath through the cuvette. Air from the OTC was continuously flushed through each cuvette. Instaneous measurements of net photosynthetic rates (P_N_) and stomatal conductance (*g*_*s*_) were carried out on the same sections of leaves after the heat treatments ended at 12:00 pm. Leaf samples collected immediately after gas exchange measurements were used to determine electrolyte leakage, or frozen in liquid nitrogen for further analysis.

### Relative leaf injury measurements

Relative leaf injury (*R*_I_) was measured by quantifying electrolyte leakage before and after heat stress treatments, as described previously [14].

### Quantitative transcript abundance

Changes of transcript abundance (qPCR) in maize leaves were determined as described previously [27]. Two maize leaf dics (0.6 cm diameter, and approximately 0.5 g fresh weight) were ground using liquid N_2_ in a sterile mortar and pestle, and total RNA was extracted using TRIzol® reagent according to the manufacturer’s instructions (Invitrogen, Carlsbad, CA). After quantification with a NanoDrop spectrophotometer (model 2000c, Thermo-Fisher Scientific Inc., Waltham, MA), first strand cDNA was synthesized with 2 μg of total RNA (OD260 nm/OD280 nm > 1.95) using oligo(dT) 20 primers and SuperScript III RNase H reverse transcriptase from Invitrogen. The resultant cDNA was diluted 10-fold and was used as a template for real-time quantitative polymerase chain reaction (qPCR). Amplifications were performed with a model Mx3005P qPCR System plus Brilliant SYBR® Green qPCR Master Mix (Stratagene, La Jolla, CA).

Primers and functional annotations for C_4_ related photosynthetic enzyme genes are listed in Table 1. Assays were performed with three biological samples from each treatment, and measurements were replicated three times. Maize *ACTIN1* gene was used as an expression control and relative transcript abundance was calculated by 2^-ΔΔCT^×100 according to Pfaffl [27].

**Table 1:** Primer information about key C_4_ enzymes determined in this study.

GenBank#	Name	Sequence	Product length (bp)
HQ697600.1	*NADP-ME-F*	AGGCTCTCTTCAGCCATTCA	173
	*NADP-ME-R*	TAGGCCTCTCGTTGAAGGAA	
	*PEPC-R*	CCACCCATCCAAGAAGAGAA	
EU967073.1	*Actin1-F*	CTATGTTCCCTGGCATTGCT	188
	*Actin1-R*	GGGCCCAAAGAATTAGAAGC	

### C_4_ photosynthetic enzyme assays

Five leaf discs (about 3.14 cm^2^) were removed from the lamina of leaves in field experiments as described above. Leaf materials were rapidly transferred to labeled envelopes and immediately immersed in liquid N_2_ to quench metabolism. All samples were stored at −80°C prior to analysis. Two leaf discs from each plant were extracted with 0.6 ml ice cold extraction buffer consisting of 50 mM Tris–HCl (pH 7.50), 10 mM MgCl_2_, 1 mM EDTA, 1% (w/v) PVP-40, 5 mM Na^+^-pyruvate and 10% glycerol. Immediately prior to extraction, freshly prepared 1 μM leupeptin and 5 mM dithiothreitol were added to the buffer solution. Samples were extracted at 0°C with a ground glass tissue homogenizer and the homogenates were transferred to 2 ml plastic centrifuge tubes and spun for 3 min at 12,000 × *g* in an Eppendorf model 5415D microfuge. The supernatant was transferred to a 1.5 mL Eppendorf tube and assayed immediately or stored briefly in liquid N_2_.

Enzyme activity measurements were performed spectrophotometrically at 25°C as described by Maroco et al. [28]. Activities of NADP-malate dehydrogenase (MDH) were measured in 1 ml solution containing 50mM Tris-HCl (PH 8.0), 1 mM EDTA, 100 mM oxalacetic acid, 10 mM NAHPH and 0.025 ml leaf extract. PEP carboxylase (PEPCase) activities were measured in 1 ml solution containing 50 mM Tris–HCl (pH 8.0), 5 mM NaHCO_3_, 5 mM MgCl_2_, 0.14 mM NADH, 10 mM PEP (tricyclohexlamine salt), 1 unit Malate dehydrogenase and 0.025 ml sample as described in Ziska et al. (1999). Activities of NADP-malic enzyme (NADP-ME) were measured in 1 ml solution containing 50 mM Tris-HCl (pH 8.0), 5mM EDTA, 500 mM MgCl_2_, 100 mM malic acid, 250 mM dithioerythritol, 20 mM NADP^+^ and 0.025 ml sample. All measurements were performed using a Shimadzu model 2101 spectrophotometer operated in the kinetic mode. Enzyme activities were calculated from the rate of the changes in optical wavelength at 340 nm.

### GC-MS measurements

Freeze-dried leaf tissue (~30 mg total) for each treatment was added to a 2.0 ml Eppendorf tube containing a 3.2 mm ceramic bead and ~100 μl fine garnet powder. Maize leaf tissue was homogenized in a Tissue Lyzer ball mill at 30 cycles s^-1^. A 50 μl mixture of 2.5 mM *α*-aminobutyric acid, 2.0 mg ribitol and 1.4 ml ice-cold 70% methanol was injected into each sample and vortexed vigorously. The suspended plant tissues were heated to 45°C for 15 minutes in a water bath and then the extracts were microcentrifuged for 5 minutes at 12000 x *g* as described above. Supernatants were gently transferred into 15 ml fresh conical, plastic centrifugation tubes. The pellets were washed once with 70% methanol as described above and the supernatants were combined. The washed pellets were air-dried overnight and used for the determination of starch as previously described [29].

A total of 10 organic acids and soluble carbohydrates was measured by gas chromatography coupled to mass spectrometry (GC-MS) as described by Roessner et al. [30]. Derivative samples were separated by gas chromatography and the resultant ions were detected with a mass selective detector (model 7125, Agilent technologies, Wilmington, DE). Total ion chromatograms were quantified using peak identification and calibration parameters within the Agilent MSD Chemstation software program. Independent standard curves were prepared for each set of extractions with known mixtures of organic acids and soluble carbohydrates. Ribitol was added during extraction functioned as the internal standard. Compounds included in the organic acid fraction were citrate, aconitate, malate, fumarate and succinate. Compounds in the soluble carbohydrate fraction were fructose, glucose, sucrose, maltose, and starch.

### Statistical analysis

Each gas exchange analysis in OTC experiment is the mean of 11 independent measurements. For PEPCase assays, membrane integrity and qPCR, data was derived from 3 independent replicates for each maize cultivar. One-way analysis of variance (ANOVA) via software SPSS 10.0 (SPSS Inc., USA) was applied to identify significant differences between heat stress and CO_2_ treatments for specific maize cultivars or for specific daily time point. Three-way ANOVA using R software (3.3.0 version) was used to test the significant effects of maize cultivars, grown CO_2_ and heat stress treatments, and their interactions on physiological traits and metabolites.

## Results and analysis

### Differential response of relative leaf injury to heat stress in two maize cultivars

**Fig 1:**
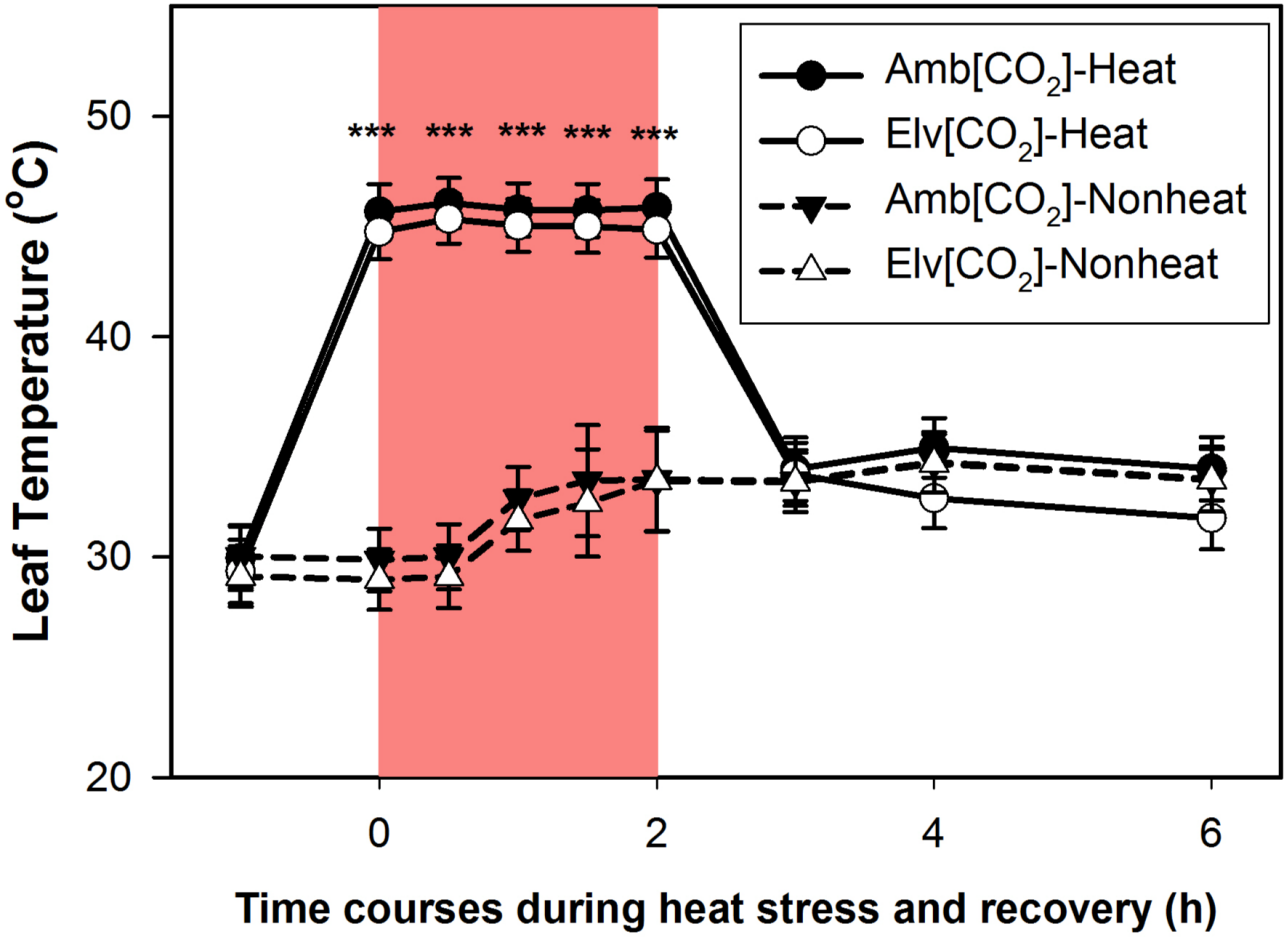
Time courses of leaf temperature in response to combinations of grown CO_2_ and sudden heat shock treatments. There are four combinations: grown ambient CO_2_ with heat treatments (Amb[CO_2_]-Heat), grown elevated CO_2_ with heat treatments (Elv[CO_2_]-Heat), grown ambient CO_2_ without heat treatments (Amb[CO_2_]-Nonheat), grown elevated CO_2_ without heat treatments (Elv[CO_2_]-Nonheat). The abbreviations for combinations of CO_2_ and heat treatments were same as following figures. Red regions represent 2 hours sudden heat shock treatments. One-way ANOVA was applied to analyze statistical significance levels of leaf temperature among the four combinations for each time point, while symbol “***” represent *P*-value <0.001.

Mean temperatures of non-heat treated leaves were about 31°C when measured at 10:00 am and this increased about 1.5°C during the 2 h period that sudden heat shock (SHS) was applied (Fig 1). The increases in leaf temperature between 10:00 am and 12:00 pm can be attributed to natural, diurnal temperature fluctuations in the field. Temperatures across heat treated and non-heat treated leaves did not differ significantly between the CO_2_ treatments at any time (Fig 1).

**Fig 2:**
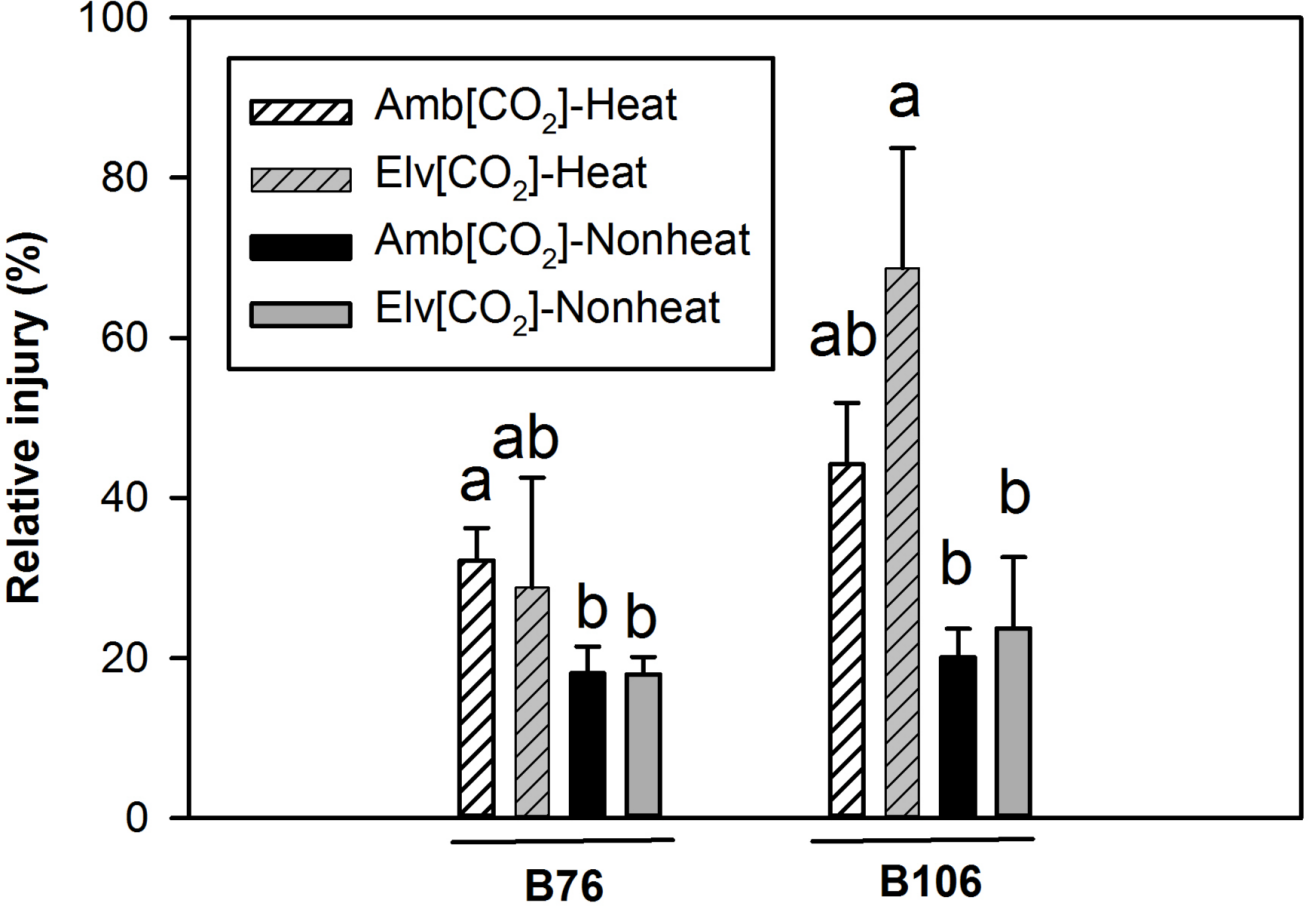
Relative injury (*R*_I_) in response to sudden heat shock of two maize cultivars grown under ambient and elevated [CO_2_]. Within each cultivar, values of different CO_2_ and heat treatments with same letter are not significantly different (*P*<0.05) based on one-way ANOVA analysis. Vertical bars represent S.E. for n = 3.

Differences in relative leaf injury (*R*_I)_ among the two maize inbred lines due to SHS are shown in Fig 2 and Table 2. The highest values of *R*_I_ due to SHS were observed for B106 inbred line, with 70% regarding elevated [CO_2_]–nonheat treated leaves. In contrast, *R*_I_ values of B76 inbred line were significantly unaffected by SHS across [CO_2_] treatments. In contrast to SHS treatments, CO_2_ treatments did not significantly alter *R*_I_ in B76.

**Table 2:** Results from statistical analyses (*P* values) for treatments effects in physiological traits and metabolites. Ns: not significant.

	Cultivar	CO_2_	Heat	Cultivar×CO_2_	Cultivar×Heat	CO_2_×Heat	CO_2_×Heat×Cultivar
P_N_	<0.001	<0.001	<0.001	<0.001	ns	ns	<0.001
*g*_*s*_	ns	<0.001	<0.001	<0.001	<0.001	ns	ns
*R*_I_	<0.001	ns	<0.001	ns	ns	ns	ns
PEPCase	ns	0.04	<0.001	ns	ns	ns	ns
NADP-ME	ns	ns	<0.001	ns	<0.001	<0.001	<0.001
NADP-MDH	0.03	ns	<0.001	ns	ns	ns	ns
*PEPC*	<0.001	<0.001	0.04	0.03	ns	ns	ns
*NADP-ME*	ns	ns	<0.001	ns	ns	ns	ns
*NADP-MDH*	0.05	ns	<0.001	0.03	0.01	ns	ns
Starch	<0.001	<0.001	<0.001	<0.001	<0.001	<0.001	<0.001
Maltose	ns	ns	0.03	ns	ns	ns	ns
Sucrose	ns	ns	0.02	ns	ns	ns	ns
Glucose	ns	ns	<0.001	ns	ns	ns	ns
Fructose	ns	ns	0.01	ns	ns	ns	ns
Succinate	<0.001	<0.001	<0.001	ns	<0.001	ns	ns
Fumarate	<0.001	ns	0.04	ns	ns	ns	ns
Malate	<0.001	ns	<0.001	ns	<0.001	ns	<0.001
Aconitate	<0.001	ns	0.03	<0.001	<0.001	ns	<0.001
Citrate	<0.001	ns	<0.001	<0.001	<0.001	<0.001	ns

### Responses of leaf gas exchange to heat stress and varying CO_2_ concentration

**Fig 3:**
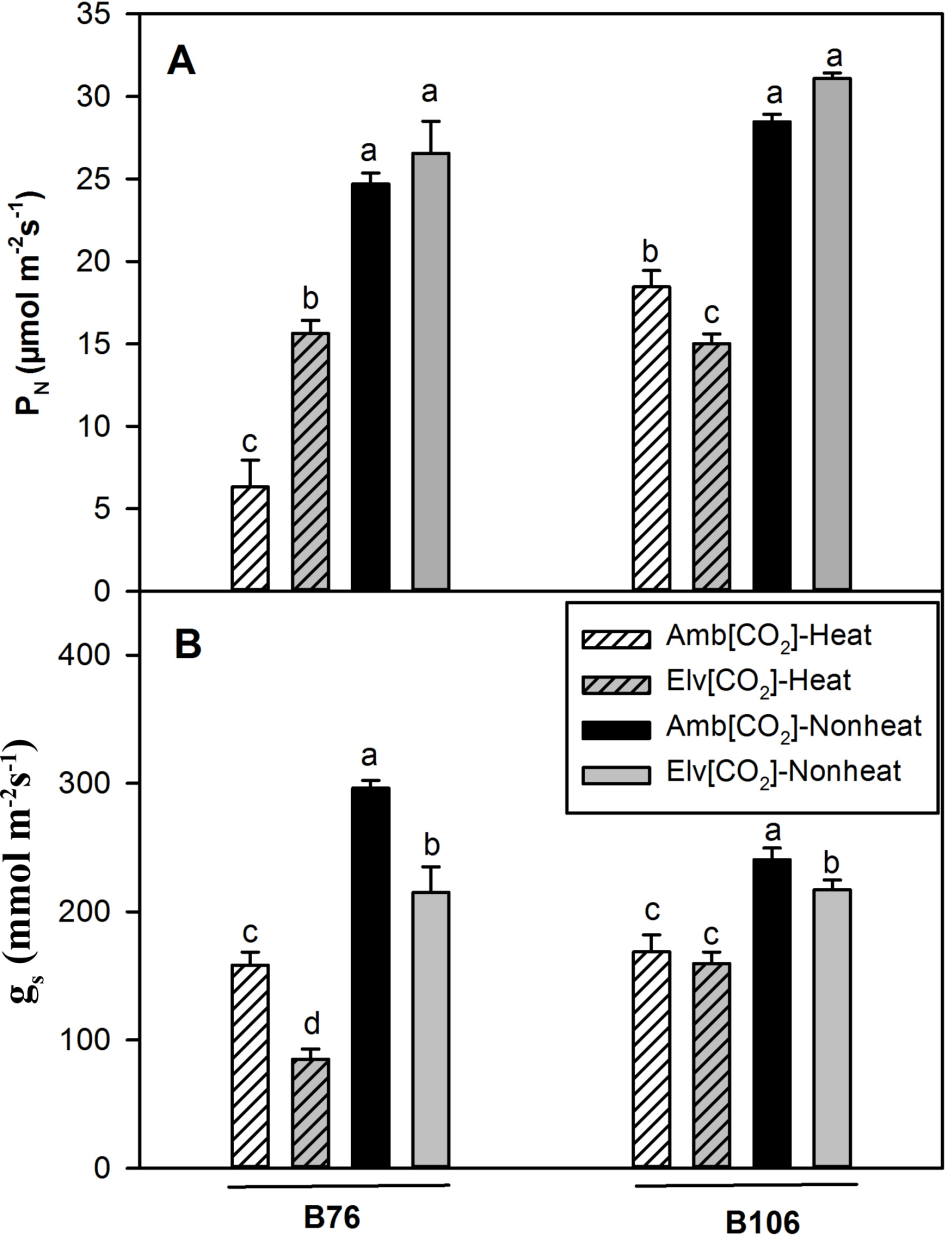
Photosynthetic rates (P_N_) and stomatal conductance (*g*_*s*_) in response to a 2 hour sudden heat shock treatment in B76 and B106 grown under ambient or elevated [CO_2_]. Within each cultivar, values of different CO_2_ and heat treatments with same letter are not significantly different (*P*<0.05) based on one-way ANOVA analysis.

Effects of heat stress, CO_2_ and cultivars, and their interactions on P_N_ were significant (Table 2). Rates of P_N_ for both maize inbred lines were between 24.7 and 30.9 μmol m^- 2^ s^-1^ for nonheat treated leaves in either CO_2_ treatment (Fig 3A). Rates of P_N_ in B76 decreased about 75% under ambient CO_2_ following 2 h SHS compared with nonheat treated leaves. In terms of B76 grown under elevated [CO_2_], SHS dependent decrease of P_N_ is about 41% compared with nonheat treatments. SHS inhibited P_N_ in B106 about 35% and 51% in the ambient and elevated [CO_2_] treatments, respectively.

Values of *g*_*s*_ for B76, immediately after SHS treatment at elevated CO_2_ were about 60% lower than that of elevated [CO2] with nonheat treatments (Fig 3B). In comparison with nonheat treatments, the reduction of *g*_*s*_ due to SHS for B106 was no more than 35% across CO_2_ treatments. As expected, enhanced CO_2_ treatments decreased *g*_*s*_ of both cultivars majorly due to CO_2_ induced stomatal closure.

### Responses of C_4_ enzyme activities to heat stress and CO_2_ enrichment

**Fig 4:**
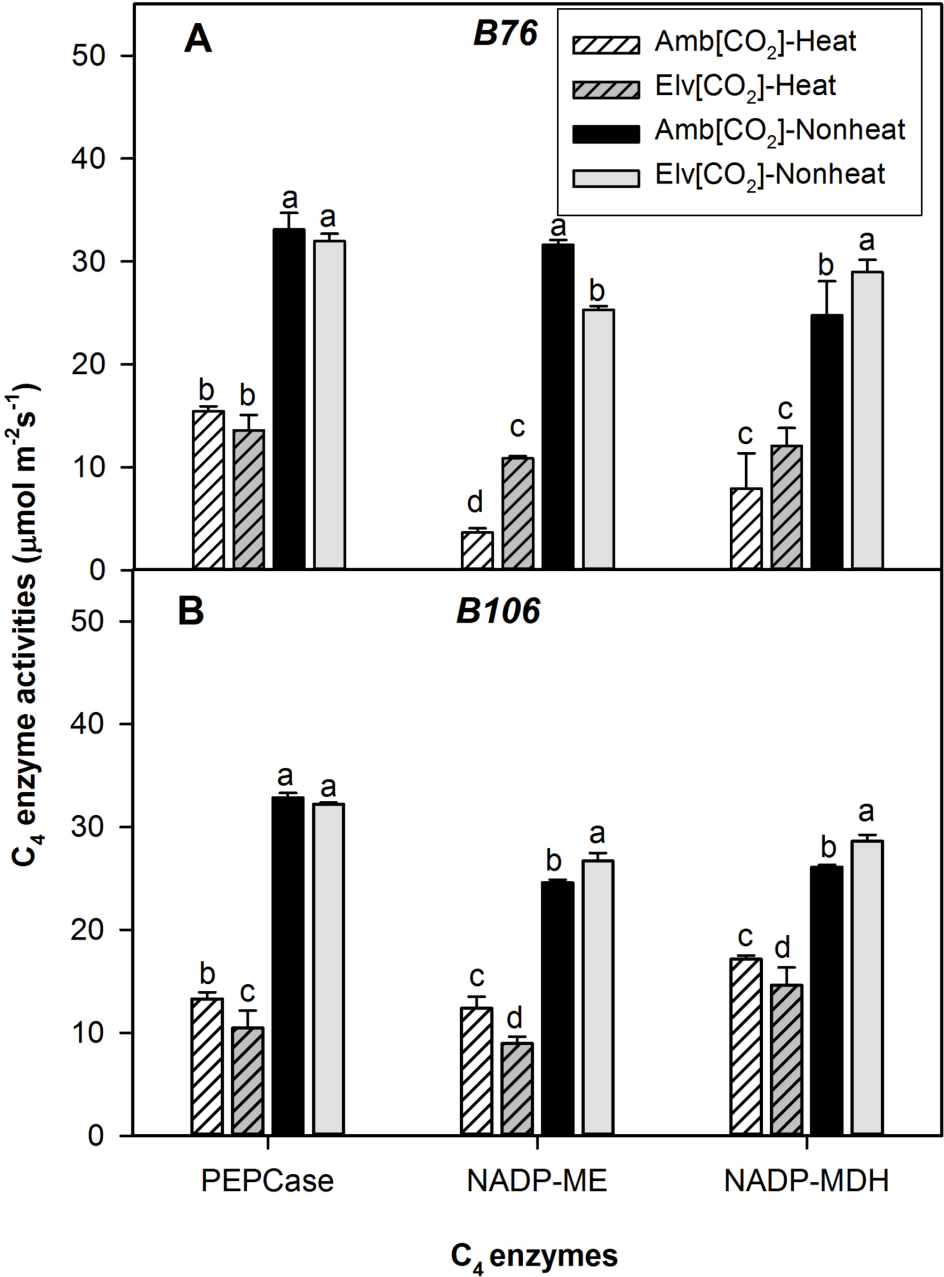
*In vitro* activities of C_4_ assimilation pathway enzymes in response to sudden heat shock treatments in B76 and B106 grown under ambient or elevated [CO_2_]. Within each cultivar, values of different CO_2_ and heat treatments with same letter are not significantly different (*P*<0.05) based on one-way ANOVA analysis. Vertical bars represent S.E. for n = 3.

The enzymes, PEPCase, NADP-ME and NADP-MDH, function in the C_4_ dicarboxylic acid cycle and catalyze important photosynthetic reactions in maize. Activities of all three C_4_ enzymes in both maize inbred lines significantly decreased in response to SHS treatments (Fig 4; Table 2). Activities of NADP-ME in the ambient [CO_2_] treatment in B76 were decreased 88% by SHS compared with nonheat treatments, and this is the greatest reduction of enzyme activity due to SHS. Conversely, activities of NADP-MDH in ambient [CO_2_] treatment were only reduced 34% by SHS. Effects of elevated [CO_2_] on the response of C_4_ cycle enzyme activities in both maize inbred lines during SHS were negligible and inconsistent. When comparing averaged reduction of all three enzyme activities across combined treatments, it is obvious that SHS effects were greatest for the B76 genotype and were least for the B106 genotype when comparing experiments performed in ambient [CO_2_] treatments (Fig 4).

### Transcript expression of C_4_ enzymes in response to heat stress and CO_2_ enrichment

**Fig 5:**
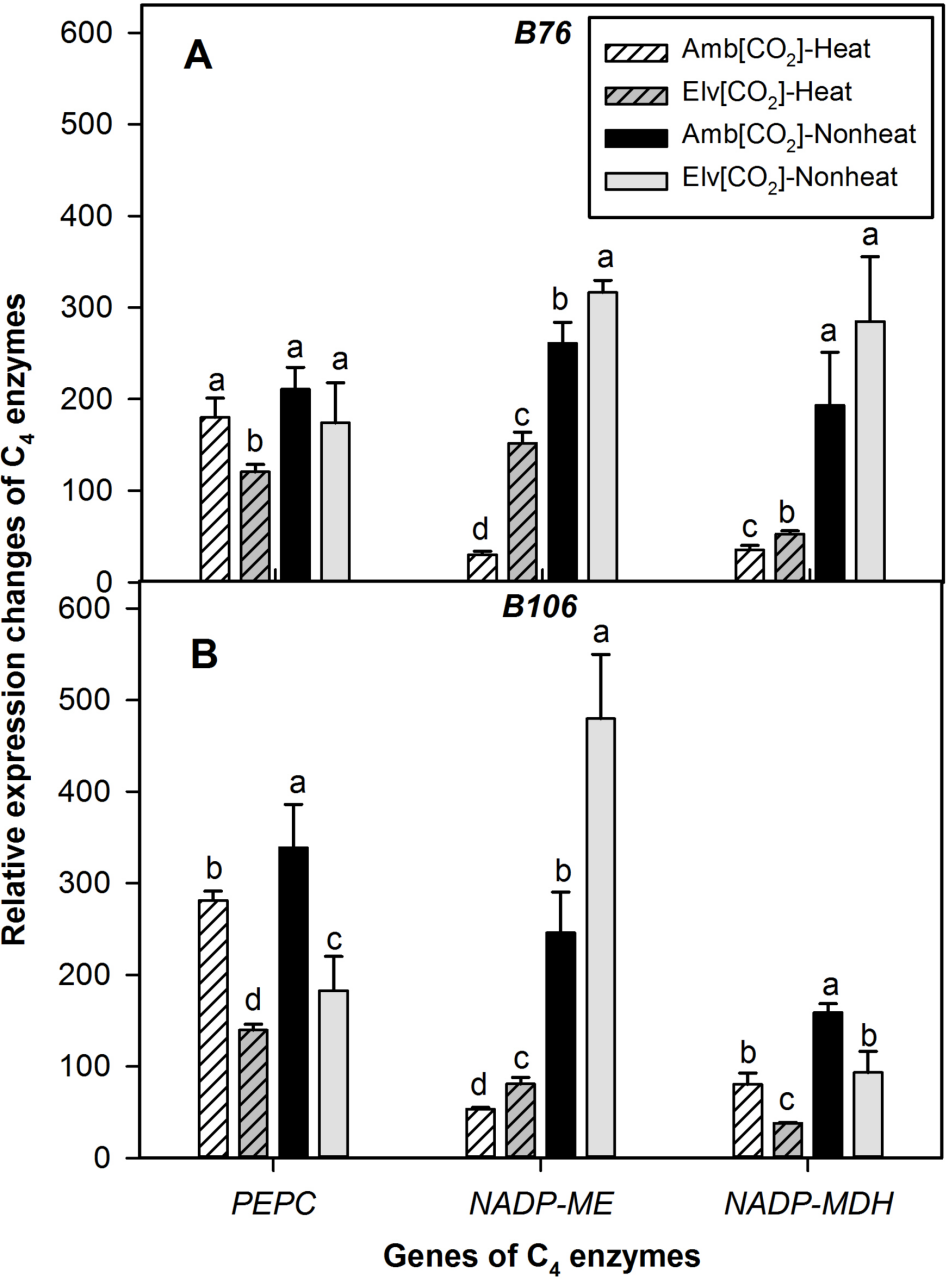
Relative transcript abundance of C_4_ assimilation pathway genes in response to sudden heat shock treatments in B76 and B106 grown under ambient or elevated [CO_2_]. Within each cultivar, values of different CO_2_ and heat treatments with same letter are not significantly different (*P*<0.05) based on one-way ANOVA analysis. Vertical bars represent S.E. for n = 3.

Changes of transcript expression in response to SHS and CO_2_ treatments were also determined for the three C_4_ enzymes as described above (Fig 5). Overall, the expression of *PEPC* gene decreased from 14 to 31% in response to SHS compared with nonheat stress treatments in either ambient or elevated [CO_2_] across two maize genotypes. In contrast, the expression of *NADP-ME* gene decreased over 80% in both maize inbred lines and in either CO_2_ treatment. The expression of NADP-ME in B76 grown under ambient [CO_2_] was reduced 88% for SHS treated leaves relative to nonheat treated leaves. The inhibition effects of SHS on the expression levels of *NADP-MDH* also were in excess of 80% in B76, while the expression levels decreased around 50% in B106 across CO_2_ treatments. The gene expression of *PEPC* in either genotype and in both temperature treatments was decreased 33% on average by elevated [CO_2_] relative to ambient [CO_2_]. Conversely, the effects of SHS on the expression of *NADP-ME* were greater in ambient [CO_2_] compared to elevated [CO_2_] treatment. Consistent effects of CO_2_ enrichment were not observed for changes of expression of *NADP-MDH* due to SHS.

### Effects of heat stress and CO_2_ enrichment on soluble metabolite concentrations in maize leaves

**Fig 6:**
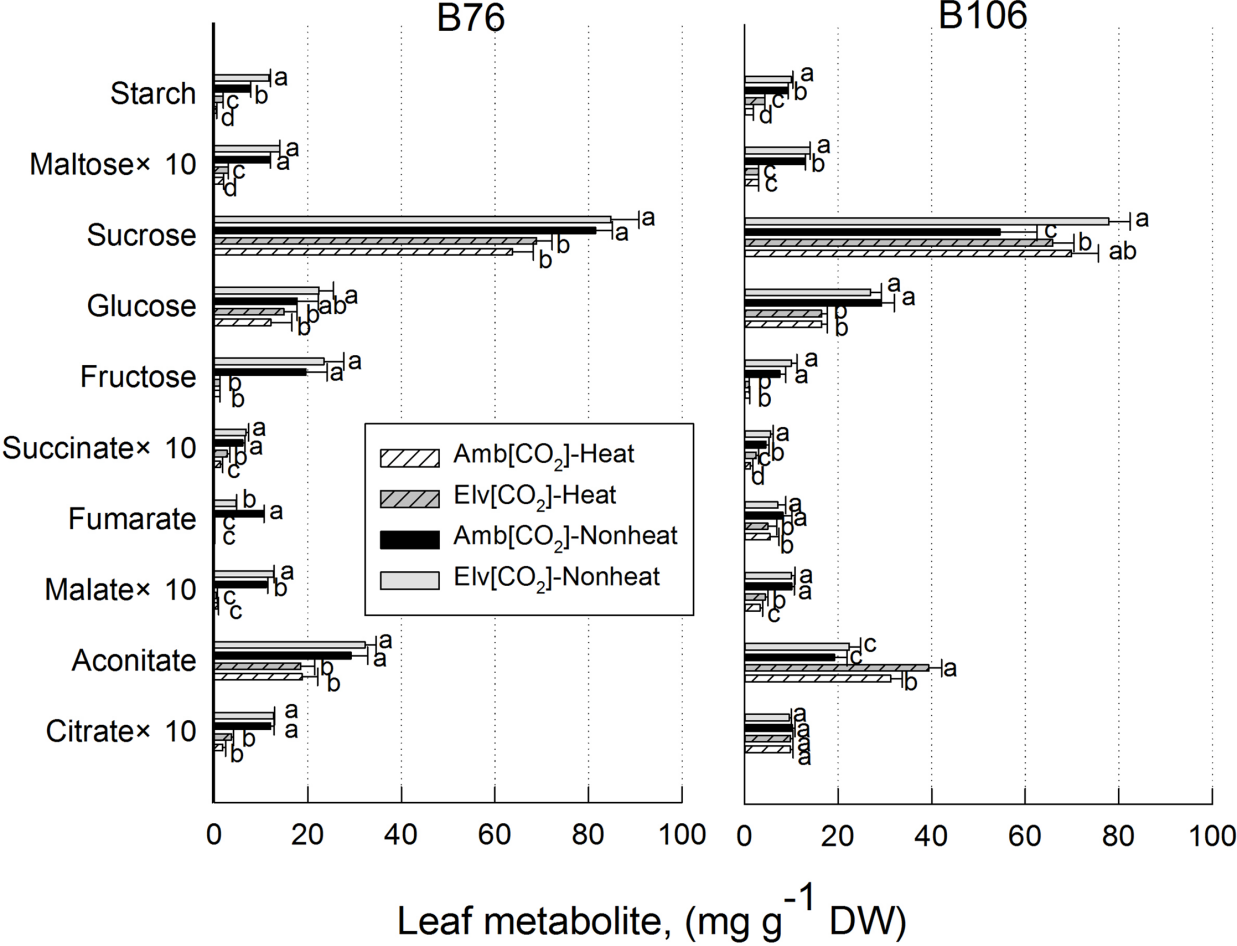
Effects of sudden heat shock treatments on maize leaf metabolites in B76 and B106 grown under ambient and elevated [CO_2_]. Values for some metabolites were multiplied by 10 for clarity, as indicated by the axis label. Within each metabolite, values of different CO_2_ and heat treatments with same letter are not significantly different (*P*<0.05) based on one-way ANOVA analysis. Vertical bars represent S.E. for n = 3.

Pronounced changes of maize leaf metabolites occurred in response to SHS treatments in both maize genotypes used in this study (Fig 6; Table 2). The effects of either maize inbred lines, grown CO_2_ and SHS treatments, or their interactions on starch were significant. (Table 2). Leaf starch levels decreased by 60 to almost 100% in both maize inbred lines following 2 h SHS. In contrast to starch, glucose levels across CO_2_ treatments increased 3 ~ 8 fold and almost 2 fold due to SHS in B76 and B106, respectively. Both maltose and sucrose decreased in response to SHS in B76 and in particular, maltose was reduced over 70%. Similar results for maltose and sucrose were observed for the B106 except that sucrose slightly increased in response to SHS in the ambient CO_2_ treatment. Other than starch there was no evidence that elevated CO_2_ treatments influenced soluble carbohydrate concentrations in this study (Fig 6; Table 2).

The SHS treatments also exerted dramatic effects on organic acids associated with either the TCA cycle or with the C_4_ carbon assimilation metabolism in maize leaves. Citrate, aconitate, malate, fumarate and succinate were decreased by SHS in B76. Both citrate and malate decreased 70% or more in B76 in response to SHS. These organic acids in B106 were also decreased by SHS, but the values of reductions by SHS were less than that observed in B76. Interestingly, SHS treatments did not affect citrate levels in B106 across CO_2_ treatments. Malate levels were 5 to 10 fold higher in B106 compared to B76 when measurements were made following SHS across CO_2_ treatments. There were no consistent effects of CO_2_ enrichment on organic acids levels in this study (Fig 6; Table 2).

## Discussion

### Differential heat-response of electrolyte leakage in two maize genotypes

Chen et al. [24] observed substantial differences in leaf damage due to high temperatures stress under both field and controlled environment conditions when various maize inbred lines were screened for abiotic stress tolerance. In particular, visible differences in leaf damage were observed between two inbred lines B76 and B106, after intact plants were exposed to 37 to 39 ^o^C maximum air temperatures for 1 to 2 d in the field. These findings were confirmed when 38/30 ^o^C growth treatments were applied to the same maize inbred lines in a greenhouse study [24]. In the current field study, open-top chambers (OTCs) were applied to evaluate physiological and metabolic response of the two maize lines to a 2 h sudden heat shock (SHS) treatments under different grown CO_2_ conditions. Compared with FACE, OTCs remain a workable alternative with relatively stable CO_2_ control, simple technical requirements and economize benefits [31]. We observed that relative injury as assayed by electrolyte leakage was greater in B106 than in B76 following the SHS treatments (Fig 2). Similar results were observed when these experiments were repeated in controlled environment chambers (not shown). The above findings suggested that B76 exhibited greater tolerance of heat stress than B106 and were in agreement with results published by Chen et al. [24]. Our previous study [14] on a different maize line demonstrated that more ion leakage was observed at elevated than at ambient CO_2_ in response to 45 ^o^C heat stress. This was also found in the present study for maize inbred line B106, but not for B76.

### Photosynthetic response to heat stress under elevated CO_2_

In a prior study [4], we hypothesized that elevated [CO_2_] could protect photosynthesis (P_N_) from high temperature treatments by decreasing stomatal conductance (*g*_*s*_) and transpiration, and improve the leaf water use efficiency. However, in that study, the 45 ^o^C high temperature treatments eliminated CO_2_ concentration effects on *g*_*s*_ in one maize inbred line. In current study, using two maize cultivars, B76 and B106 with contrasting heat tolerance, we found that the reduction of *g*_*s*_ by elevated [CO_2_] during SHS treaments in the field did protect P_N_ of inbred line B76 (Fig 3). In contrast, there was much less reduction in *g*_*s*_ in B106 at elevated CO_2_ and thus no protection of P_N_ by elevated CO_2_ in response to SHS treatments.

### Metabolite responses to heat shock

In this study, gas chromatography–mass spectrometry (GC/MS)-based metabolomics profiling method was used, since GC-MS has very high sensitivity and can therefore be used for the analysis of less commonly encountered types of samples [32]. Metabolite changes in response to SHS were similar to prior findings for soybean plants that were grown at 8°C above ambient temperatures [33]. First, transitory starch in maize leaves was diminished by exposure to SHS. This likely represented the mobilization of stored starch rather than decreases of P_N_. CO_2_ stimulates starch accumulation for both heat stress and non-heat stress leaves, and the interactions of CO_2_ and SHS on starch were significant (Table 2). Glucose accumulated 2 to 8 fold in response to SHS in the leaves of both maize inbred lines and this result differed from soybean leaflets in which hexose levels were unaffected by elevated growth temperatures [33]. The accumulation of glucose in maize leaves in response to drought was attributed previously to the induction of a specific vacuolar acid invertase [34] and it is possible that a similar mechanism functions during SHS in the current study. The transformation of starch into glucose would be expected to boost the osmotic potential of leaves exposed to abiotic stress and this would be favorable for stress tolerance [35]. Fructose and sucrose are major end products of photosynthesis in plants. Sucrose decreased in three of four instances in this study (Fig 6), and this compound also decreased 20 to 30% in heat treated soybean leaves [33]. Also, results for fructose varied in this study. Foliar concentrations of this reducing sugar increased in B76 and decreased in B106 in response to SHS under both CO_2_ treatments (Fig 6). Overall, all of the major carbohydrates in this study decreased in genotype B76 after SHS treatments, except glucose. In comparison, fructose, glucose and in one instance sucrose increased in genotype B106.

All of the organic acids in this study decreased in B76 leaves, and in particular, some organic acids, such as citrate and malate, decreased over 70% due to SHS (Fig 6). The organic acids measured in this study were all associated with the TCA cycle and our findings suggested that TCA cycle activity was suppressed by SHS treatments in B76. The results were consistent with the evidence observed in soybean leaflets grown at high temperatures [33] and with prior observations involving abiotic stress effects on respiration [36]. However, large genotypic differences were observed in current study in regard to SHS effects on leaf metabolites. Citrate was unaffected by SHS in B106 and aconitate increased almost 2 fold in response to SHS in this genotype. Also the effects of SHS on malate, fumarate and succinate levels in B106 were less than that was observed in B76 (Fig 6). Taken together, down regulation of the TCA cycle due to SHS treatments was less severe in B106 compared to B76 (Fig 6), and interactive effects of CO_2_ and SHS on chosen compounds in TCA cycle were not significant except for citrate (Table 2).

### Disruption of C_4_ carbon assimilation cycle by heat stress

Law and Crafts-Brandner [10] suggested that the primary site of high-temperature inhibition of P_N_ in maize leaves was decreased ribulose-1,5-bisphophate carboxylase/oxygenase (Rubisco) activity and activities of Rubisco can be inhibited by temperatures above 40°C. However, studies on C_4_ carbon assimilation metabolism in response to heat stress were less reported. Previous study suggested that the C_4_ carbon assimilation cycle can be inhibited by water stress [8], but the question that if heat stress is able to inactivate this pathway as well remains unclear. In the present study, our findings demonstrated that relevant components of the C_4_ photosynthetic pathway were inactivated by heat stress (Figs 4 and 5). The three C_4_ enzyme activities measured in this study were inhibited a minimum of 34% by SHS treatments in both inbred lines (Fig 4). Both transcript abundance and enzyme activities of NADP-ME in B76 decreased over 85% by SHS in ambient [CO_2_] treatment (Figs 4 and 5). This enzyme is important in the conversion of malate to phospho-(enol) pyruvate, which is the substrate for PEPCase and is vital for CO_2_ fixation. Overall, the decreases of gene expression for *NADP-MDH* and *NADP-ME* in response to SHS were greater than for *PEPC* in both maize inbred lines. Malate is synthesized from oxalacetic acid in reactions catalyzed by NADP-MDH. The dramatic reductions of malate in response to SHS discussed above confirmed that the C_4_ photosynthetic pathway was inhibited by the SHS treatments imposed in this study (Figs 4 and 5). The magnitude of the reduction in C_4_ enzyme activities closely matched the reductions in P_N_ for both lines and CO_2_ treatments, suggesting that the inhibition of the C_4_ cycle was more important than disruption of the C_3_ photosynthetic pathway for this SHS. In contrast, for water stress treatments, P_N_ yielded relatively more inhibition by SHS than were C_4_ enzyme activities in these same maize lines [37].

## Conclusions

The relative injury measurements taken immediately after sudden heat shock (SHS) treatments were performed in this field study confirmed that maize inbred line B76 was more thermo-tolerant than B106. However, various other measurements in this study including P_N_, *g*_*s*_, C_4_ enzyme activities, transcript abundance, and metabolite analysis, consistently showed that inbred line B106 was more tolerant to SHS than B76, particularly under ambient CO_2_. These findings indicated that heat stress tolerance is a complex trait. Therefore, it may be difficult to identify biochemical, physiological or molecular markers that accurately and consistently predict heat stress tolerance.

## References

[1] Song Y, Yu J, Huang B. Elevated CO_2_-mitigation of high temperature stress associated with maintenance of positive carbon balance and carbohydrate accumulation in *Kentucky Bluegrass*. PLoS ONE. 2014; 9 e89725 doi: 10.1371/journal.pone.0089725 24662768 10.1371/journal.pone.0089725 PMC3963838

[2] Sage RF. Acclimation of photosynthesis to increasing atmospheric CO_2_-the gas-exchange perspective. Photosynth. Res. 1994; 39: 351–368. doi: 10.1007/BF00014591 24311129 10.1007/BF00014591

[3] Ainsworth EA, Davey PA, Bernacchi CJ, Dermody OC, Heaton EA, Moore DJ etal A meta-analysis of elevated CO2 effects on soybean (Glycine max) physiology, growth and yield. Glob. Chang. Biol. 2002; 8: 695–709.

[4] Wang D, Heckathorn SA, Barua D, Joshi P, Hamilton EW, LaCroix J. Effects of elevated CO_2_ on the tolerance of photosynthesis to acute heat stress in C_3_,C_4_, and CAM species. Am. J. Bot. 2008; 95: 165–176. doi: 10.3732/ajb.95.2.165 21632342 10.3732/ajb.95.2.165

[5] Reich PB, Tilman D, Craine J, Ellsworth D, Tjoelker M, Mark G et al Do species and functional groups differ in acquisition and use of C, N and water under varying atmospheric CO_2_ and N availability regimes? A field test with 16 grassland species. New Phytol. 2001; 150: 435–448.

[6] Morison JIL, Lawlor DW. Interactions between increasing CO_2_ concentration and temperature on plant growth. Plant Cell Environ. 1999; 22: 659–682.

[7] Sage RF, Monson RK (1999). C_4_ Plant Biology. Academic Press, San Diego

[8] Heckathorn SA, Downs CA, Sharkey TD, Coleman JS. The small, methionine-rich chloroplast heat-shock protein protects photosystem II electron transport during heat stress. Plant Physiol. 1998; 116: 439–444. 9449851 10.1104/pp.116.1.439 PMC35186

[9] Heckathorn SA, Ryan SL, Baylis JA, Wang JA, Hamilton EW, Cundiff L etal In vivo evidence from an Agrostis stolonifera selection genotype that chloroplast small heat-shock proteins can protect photosystem II during heat stress. Funct. Plant Biol. 2002; 29: 933–944. 10.1071/PP01191 32689544

[10] Law RD, Crafts-Brandner SJ. Inhibition and acclimation of photosynthesis to heat stress is closely correlated with activation of ribulose-1,5-bisphosphate carboxylase/oxygenase. Plant Physiol. 1999; 120: 173–181. 10318695 10.1104/pp.120.1.173 PMC59249

[11] Crafts-Brandner SJ, Salvucci ME. Sensitivity of photosynthesis in a C_4_ plant, maize, to heat stress. Plant Physiol. 2002; 129: 1773–1780. doi: 10.1104/pp.002170 12177490 10.1104/pp.002170 PMC166765

[12] Hamilton EW, Heckathorn SA, Joshi P, Wang D, Barua D. Interactive effects of elevated CO_2_ and growth temperature on the tolerance of photosynthesis to acute heat stress in C_3_ and C_4_ species. Journal of Integrative Plant Biology, 2008; 50: 1375–1387. doi: 10.1111/j.1744-7909.2008.00747.x 19017125 10.1111/j.1744-7909.2008.00747.x

[13] Mearns LO, Katz RW, Schneider SH. Extreme high temperature events: changes in their probabilities with changes in mean temperature. J. Clim. Appl. Meteorol. 1984; 1601–1613.

[14] Qu MN, Bunce JA, Shi ZS. Does elevated CO_2_ protect photosynthesis from damage by high temperature via modifying leaf water status in maize seedlings? Photosynthetica. 2014; 52: 211–216.

[15] Feller U, Crafts-Brandner SJ, Salvucci ME. Moderately high temperatures inhibit ribulose-1,5-bisphosphate carboxylase/oxygenase activase-mediated activation of Rubisco. Plant Physiol. 1998; 116: 539–546. 9490757 10.1104/pp.116.2.539 PMC35111

[16] Crafts-Brandner SJ, Law RD. Effect of heat stress on the inhibition and recovery of the ribulose-1,5-bisphosphate carboxylase/oxygenase activation state. Planta. 2000; 212: 67–74. doi: 10.1007/s004250000364 11219585 10.1007/s004250000364

[17] Ghannoum O. C_4_ photosynthesis and water stress. Ann. Bot. 2009; 103: 635–644. doi: 10.1093/aob/mcn093 18552367 10.1093/aob/mcn093 PMC2707343

[18] Ripley B, Freole K, Gilbert M. Differences in drought sensitivities and photosynthetic limitations between co-occurring C_3_ and C_4_ (NADP-ME) Panicoid grasses. Ann. Bot. 2010; 105: 493–503. doi: 10.1093/aob/mcp307 20106844 10.1093/aob/mcp307 PMC2826257

[19] Murakami M, Naraba H, Tanioka T. Semmyo N, Nakatani Y, Kojima F, Ikeda T, Fueki M, Ueno A, Oh-ishi S, Kudo I. Regulation of prostaglandin E_2_ biosynthesis by inducible membrane-associated prostaglandin E_2_ synthase that acts in concert with cyclo-oxygenase-2. J. Biol. Chem. 2000; 275: 32783–32792. doi: 10.1074/jbc.M003505200 10869354 10.1074/jbc.M003505200

[20] Kaplan F, Kopka J, Haskell DW, Zhao W, Schiller C, Gatzke N, Sung DY, Guy CL. Exploring the temperature-stress metabolome of *Arabidopsis*. Pl. Physiol. 2004; 136:4159–4168. doi: 10.1104/pp.104.052142 15557093 10.1104/pp.104.052142 PMC535846

[21] Mayer RR, Cherry JH, Rhodes D. Effects of heat shock on amino acid metabolism of cowpea cells. Pl. Physiol. 1990; 94: 796–810. 10.1104/pp.94.2.796 PMC1077301 16667781

[22] Lavola A, Julkunen-Tiitto R. The effect of elevated CO_2_ and fertilization on primary and secondary metabolites in birch, *Betula pendula* (Roth). Oecologia. 1994; 99: 315–321. doi: 10.1007/BF00627744 28313886 10.1007/BF00627744

[23] Chen JP, Xu WW, Burke JJ, Xin GJ. Role of phosphatidic acid in high temperature tolerance in maize. Crop Sci. 2010; 50:2506–2515.

[24] Chen J, Xu W, Velten J, Xin Z, Stout J. Characterization of maize inbred lines for drought and heat tolerance. J. Soil Water Conser. 2012; 67: 354–364.

[25] Bunce JA. CO_2_ enrichment at night affects the growth and yield of common beans. Crop Sci. 2014; 54: 1–4.

[26] Sicher RC, Barnaby JY. Impact of carbon dioxide enrichment on the responses of maize leaf transcripts and metabolites to water stress. Physiol. Plantar. 2012; 144: 238–253. 10.1111/j.1399-3054.2011.01555.x 22150442

[27] Pfaffl MW. A new mathematical model for relative quantification in real-time RT-PCR. Nucleic Acids Res. 2(2001) e45. 10.1093/nar/29.9.e45 PMC55695 11328886

[28] Maroco JW, Edwards GE, Ku MSB. Photosynthetic acclimation of maize to growth under elevated levels of carbon dioxide. Planta. 1999; 210: 115–125. doi: 10.1007/s004250050660 10592039 10.1007/s004250050660

[29] Sicher RC, Bunce JA, Growth, photosynthesis, nitrogen partitioning and responses to CO_2_ enrichment in a barley mutant lacking NADH-dependent nitrate reductase activity. Physiol. Plant. 2008; 134: 31–40. doi: 10.1111/j.1399-3054.2008.01127.x 18485057 10.1111/j.1399-3054.2008.01127.x

[30] Roessner U, Wagner C, Kopka J, Trethewey RN, Willmitzer L. Simultaneous analysis of metabolites in potato tuber by gas chromatography–mass spectrometry. J. Plant. 2000; 23: 131–142. 10.1046/j.1365-313x.2000.00774.x 10929108

[31] Fangmeier A, Stein W, Jäger J. Advantages of an open-top chamber plant exposure system to assess the impact of atmospheric trace gases on vegetation. Vereinigung für Angewandte Botanik. 1991; 66: 97–105.

[32] Fancy S-A, Rumpel K. GC-MS-Based Metabolomics In: Wang F, editor. Biomarker Methods in Drug Discovery and Development. Totowa, NJ: Humana Press; 2008 pp. 317–340. doi: 10.1007/978-1-59745-463-6_15

[33] Sicher RC. Combined effects of CO_2_ enrichment and elevated growth temperatures on metabolites in soybean leaflets: evidence for dynamic changes of TCA cycle intermediates. Planta. 2013; 238:369–380. doi: 10.1007/s00425-013-1899-8 23716183 10.1007/s00425-013-1899-8

[34] Kim JY, Mahe A, Branegon J, Prioul JP. A maize vacuolar invertase, *IVR2*, is induced by water stress. Organ/tissue specificity and diurnal modulation of expression. Pl. Physiol. 2000; 124: 71–84. 10.1104/pp.124.1.71 PMC59123 10982423

[35] Sharp RE, Poroyko V, Kejlek LF, Spollen WG, Springer GK, Bohnert HJ, Nguyen HT. Root growth maintenance during water deficits: physiology to functional genomics. J.Exp. Bot. 2004; 55: 9–19. 10.1093/jxb/erh276 15448181

[36] Atkin OK, Macherel D. The crucial role of plant mitochondria in orchestrating drought tolerance. Ann. Bot. 2009; 103: 581–597. doi: 10.1093/aob/mcn094 18552366 10.1093/aob/mcn094 PMC2707344

[37] Sicher R, Bunce J, Barnaby J, Bailey B. Water-deficiency effects on single leaf gas exchange and on C_4_ pathway enzymes of maize genotypes with differing abiotic stress tolerance. Photosynthetica. 2015; 53: 3–10. doi: 10.1007/s11099-015-0074-9

